# Supplementary material for: Predictive Model of Gemtuzumab Ozogamicin Response in Childhood Acute Myeloid Leukemia on Event-Free Survival: Data Analysis Based on Trial AAML0531
Source: Bioengineering (Basel). 2025 Mar 14;12(3):297. doi: 10.3390/bioengineering12030297 (PMC11939501; doi:10.3390/bioengineering12030297)
Supplement: Supplementary file 1 [file bioengineering-12-00297-s001.zip › Supplementary Figure S2.pdf]

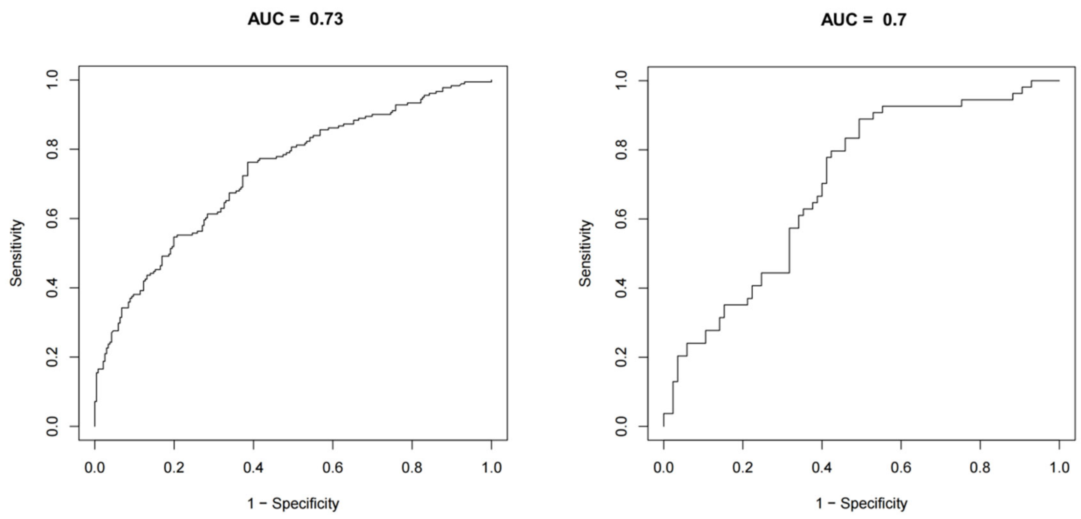

(**A**) (**B**)

Supplementary Figure S2. Receiver operator characteristic (ROC) curves of the nomogram. (**A**) ROC curves of the nomogram in the developing group. (**B**) ROC curve of the nomogram in the validation group.
