# Supplementary material for: Predictive Model of Gemtuzumab Ozogamicin Response in Childhood Acute Myeloid Leukemia on Event-Free Survival: Data Analysis Based on Trial AAML0531
Source: Bioengineering (Basel). 2025 Mar 14;12(3):297. doi: 10.3390/bioengineering12030297 (PMC11939501; doi:10.3390/bioengineering12030297)
Supplement: Supplementary file 1 [file bioengineering-12-00297-s001.zip › Supplementary Table S1.pdf]

Supplementary Table S1. AAML0531 Therapeutic Regimen

Course and drugs	Does	Days
Induction Course Ⅰ		
Cytarabine	100 mg/m^2/dose twice per day IV	1-10
Daunomycin	50 mg/m^2/dose IV	1, 3, 5
Etoposide	100 mg/m^2/dose IV	1-5
Gemtuzumab Ozogamicin	3 mg/m^2/dose IV over 2 hours	6
Induction Course Ⅱ		
Cytarabine	100 mg/m^2/dose twice per day IV	1-8
Daunomycin	50 mg/m^2/dose IV	1, 3, 5
Etoposide	100 mg/m^2/dose IV	1-5
Intensification course Ⅰ		
Cytarabine	1,000 mg/m^2/dose twice per day IV	1-5
Etoposide	150 mg/m^2/dose IV	1-5
Intensification course Ⅱ		
Mitoxantrone	12mg/m^2/dose IV	3-6
Cytarabine	1,000 mg/m^2/dose twice per day IV	1-4
Gemtuzumab	3 mg/m^2/dose IV over 2 hours	7
Intensification course Ⅲ		
Cytarabine	3,000 mg/m^2/dose twice per day IV	1, 2, 8, 9
Escherichia coli L-asparaginase	6,000 U/m^2/dose IM	2, 9

Abbreviations: IM, intramuscular; IV, intravenous.
